# Impact of Dietary Supplementation with *Moringa oleifera* Leaves on Performance, Meat Characteristics, Oxidative Stability, and Fatty Acid Profile in Growing Rabbits

**DOI:** 10.3390/ani11020248

**Published:** 2021-01-20

**Authors:** Shaimaa Selim, Mahmoud F. Seleiman, Mohamed M. Hassan, Ahmed A. Saleh, Mohamed A. Mousa

**Affiliations:** 1 Department of Nutrition and Clinical Nutrition, Faculty of Veterinary Medicine, Menoufia University, Shibin El-kom 32514, Egypt; 2 Plant Production Department, College of Food and Agriculture Sciences, King Saud University, P.O. Box 2460, Riyadh 11451, Saudi Arabia; mseleiman@ksu.edu.sa; 3 Department of Crop Sciences, Faculty of Agriculture, Menoufia University, Shibin El-kom 32514, Egypt; 4 Department of Biology, College of Science, Taif University, P.O. Box 11099, Taif 21944, Saudi Arabia; m.khyate@tu.edu.sa; 5 Department of Poultry Production, Faculty of Agriculture, Kafrelsheikh University, Kafrelsheikh 33516, Egypt; ahmed.saleh1@agr.kfs.edu.eg; 6 Department of Nutrition and Clinical Nutrition, Faculty of Veterinary Medicine, Sohag University, Sohag 82425, Egypt; dr_m_mousa@yahoo.com

**Keywords:** growing rabbit, performance, carcass characteristics, meat quality, fatty acid profile, oxidation stability

## Abstract

**Simple Summary:**

Rabbit meat is mostly preferred by consumers owing to its high nutritive value and potential health benefits. However, rabbit meat is commonly more prone to lipid peroxidation during storage, with negative effects on quality traits of meat, due to its elevated level of unsaturation of fatty acids. *Moringa oleifera* leaves have gained great interest owing to their high nutritional value and low anti-nutritional factors. *Moringa oleifera* leaves could possibly avoid oxidation damage and exhibit antioxidant activities that can conquer free radicals and reactive oxygen species synthesis. Therefore, the objective of the current study was to evaluate the effects of feeding *Moringa oleifera* leaves on performance, carcass characteristics, antioxidant capacity, blood biochemical constituents, meat quality, and fatty acids profile of growing rabbits. *Moringa oleifera* leaves supplementation improved weight gain, feed conversion ratio, antioxidant status, and meat quality characteristics. Dietary *Moringa oleifera* leaves supplementation enhanced PUFA contents, n-3 fatty acid, crude protein, and color of meat, but lowered the relative content of ether extract of the meat. Our findings suggested that *Moringa oleifera* could be used at a level of 1.5 g/kg of the growing rabbits’ diets with beneficial impacts on performance and the nutritional value of the meat.

**Abstract:**

*Moringa oleifera* leaves (MOL) have gained great interest as a non-traditional feed ingredient due to their unique nutritional value. Therefore, the objective of the current study was to evaluate the effects of graded dietary supplementation levels with MOL on performance, carcass characteristics, antioxidant capacity, blood biochemical constituents, meat quality, and fatty acids profile of growing rabbits. A total of 120 weaned New Zealand white rabbits (6 weeks old) were randomly allotted into 4 dietary groups with 5 replicates each (*n* = 6), which were fed for 42 days with a basal diet as control or 3 experimental diets supplemented with 5, 10, or 15 g/kg MOL. The results showed that, compared to the control group, the dietary inclusion of MOL at a level of 10 and 15 g/kg DM linearly increased (*p* < 0.01) final live weight (2403.3 and 2498.2 vs. 2166.6) and average daily weight gain (36.5 and 35.51 g/d vs. 28.72 g/d), and enhanced feed conversion ratio (2.49 and 2.50 vs. 3.14). The dietary supplementation with MOL linearly increased dressing out percentage, spleen index, intestinal length, and decreased abdominal fat index (*p* < 0.01). Greater serum levels of total protein and globulin, but lower alanine aminotransferase and aspartate aminotransferase were observed in the MOL-fed rabbits (*p* < 0.01). Serum levels of total triglycerides, cholesterol, and low-density lipoprotein (*p* < 0.05) were decreased linearly and quadratically in the MOL groups compared with the control. Glutathione peroxidase activity increased (*p* < 0.01), whereas malondialdehyde decreased (*p* < 0.01) linearly and quadratically in both serum and meat, in response to dietary MOL supplementation. Dietary MOL supplementation increased the meat crude protein content but lowered the relative content of ether extract in the meat (*p* < 0.05). The relative content of the meat n-3 PUFA was increased by about 33.71%, 29.46%, and 24.36% for the MOL_0.5%,_ MOL_1%,_ and MOL_1.5%_ groups compared to control. In conclusion, MOL could be used at a level of 1.5g/kg of the growing rabbits’ diets with beneficial impacts on performance, antioxidant capacity, and the nutritional value of the meat.

## 1. Introduction

Rabbit meat is lean meat of high nutritive value, because it is rich in essential amino acids, polyunsaturated fatty acids (PUFA), vitamins, minerals [1,2], low in cholesterol contents, and does not contain uric acid compared with other meats [1,2,3]. The profitability of rabbit farms is partly depending on the effectiveness of weaned rabbits to grow healthy and to protect them from high mortality rates during the fattening period. Antibiotics are frequently used in the diets of growing rabbits because digestive disturbances are the main reason for morbidity and mortality in the rabbit industry [3].

As a consequence of the European ban on antibiotic growth promoters (AGPs) and increased consumer awareness about the consumption of healthy and safer animal products, researchers and feed companies have encouraged to seek new animal feeding approaches to substitute AGPs and synthetic antioxidants [3]. Phytogenic feed additives and/or their extracts are being progressively used in animal nutrition due to their beneficial phytochemical compounds. These active components have been shown to augment appetite, improve carcass yield, enhance digestive enzyme secretion, and stimulate an immune response, and encourage antibacterial and antioxidant properties [3,4,5]. 

*Moringa oleifera* is commonly cultivated in Africa, Asia, and the US [6]. In Egypt, *M. oleifera* is widely cultivated due to its adaptation to various environmental conditions and different types of soil. Accordingly, the leaves of *M. oleifera* are plentifully available all over the year. Recently, *M. oleifera* leaves (MOL) as animal and poultry feed supplement have gained great interest owing to their high nutritional value and low anti-nutritional factors [7,8]. Various literature on the nutrient composition of *M. oleifera* showed that their leaves are rich with essential nutrients such as protein (sulfur-containing amino acids), fatty acids (α-linolenic acid), minerals (calcium, iron, and phosphorus), vitamins (A, E, B-complex, folic acid, and ascorbic acid), and several bioactive compounds, including carotenoids, saponins, phenolics, alkaloids, and flavonoids [7,8,9]. 

The phytochemical components of MOL have been reported to have antimicrobial roles and antioxidant activities and thus, MOL is commonly used in numerous medicinal applications to control various diseases such as digestive disturbance, asthma, inflammatory disease, and cancers [10,11,12]. It has been documented that MOL could possibly avoid oxidation damage [11,12], enhance the immune response [13], exhibit antioxidant activities that can conquer free radicals and reactive oxygen species synthesis [14,15], and favorably modulating lipid metabolism [9,11] in rats. Moreover, the effective antioxidants in MOL prevent deterioration and increased the shelf life of meat products in goats [16] and poultry [17].

Previous studies have been carried out to evaluate the use of MOL as a feed supplement for poultry [18,19] and rabbits [20,21,22]. These studies concluded that MOL can be used as a good feed ingredient owing to its high nutritional value and low antinutrients level. Rabbit meat is predominantly more prone to lipid peroxidation during storage, with negative effects on the meat quality traits, owing to its elevated content of unsaturated fatty acids (USFA) [23]. To our knowledge, little information is available about the effect of dietary MOL supplementation on the meat oxidation stability, cholesterol content, and fatty acids (FA) profile of growing rabbits. Therefore, the objective of the current study was to assess the effects of graded dietary supplementation levels with MOL on performance, carcass characteristics, oxidation stability, blood parameters, meat characteristics, and FA profile of growing rabbits. The hypothesis tested was that the inclusion of MOL in the diets of growing rabbits might improve their growth performance, carcass yield, antioxidant capacity, and enrich meat with essential FA.

## 2. Materials and Methods

### 2.1. Plant Material Preparation and Analysis

MOL used in the present study were collected from a private Moringa farm in Sadat City, Menoufia, Egypt. Leaves were air-dried under shade for 5–6 days. The dried leaves were then ground using a 1-mm screen for proximate chemical analysis and through a 4-mm screen for performing pellets. Dry matter (DM), crude protein (CP), ether extract (EE), and ash of MOL were analyzed according to the procedures of the AOAC [24]. Neutral detergent fiber (NDF) and acid detergent fiber (ADF) of MOL were analyzed according to Van-Soest et al. [25]. Total phenolic contents were estimated using Folin–Ciocalteau reagent as described by Al-Farsi et al. [26] and expressed as equivalent mg gallic acid/100 g of MOL. Phenolic acids of MOL were measured in accordance with the methods of Mattila et al. [27] using high-performance liquid chromatography (HPLC). The chemical composition of MOL was shown in Table 1. 

### 2.2. Animals and Experimental Design

The experimental procedures for animal husbandry and management in the current study were permitted by the Animal Ethics Committee of Menoufia University, Menoufia, Egypt (No. 09/2018 EC). The procedures of the trial were done according to the Directive 2010/63/EU of the European Parliament and of the Council of September 22, 2010, on the protection of experimental animals. The experiment was performed at a private rabbit production farm, Menoufia, Egypt during the period from January to February 2019. Rabbits used in the present study were clinically healthy during the whole trial period. A total of 120 weaned New Zealand white rabbits (60 males and 60 females), 6 weeks old and with an average live weight of 956 ± 35.33 g was used in the experiment. Rabbits were kept in wire cage batteries with the standard dimensions of 50 cm length × 45 cm width × 40 cm height. The batteries were located 100 cm above the ground level in a well-ventilated area. The cages were supplied with nipple drinkers and feeders. All rabbits were placed under the same management, environmental, and hygienic conditions. The health status of animals was monitored throughout the experiment. Temperature and relative humidity were kept at 20 ± 3 °C and 65%, respectively. The length of daylight during the experimental period (January to the end of February) was on average 11 h. Rabbits were randomly divided into four treatment groups of 30 animals each balanced for sex (6 rabbits (3 males + 3 females) each cage × 5 replicates). The control group (CON) received a weaning-fattening basal diet without MOL, whereas the 2–4 treatment groups were fed on the MOL_0.5%_, MOL_1%_, and MOL_1.5%_ diets which contained 5, 10, and 15 g MOL per kg of diet, respectively. The experiment period lasted for 42 days. All growing rabbits were provided feed and water ad libitum. The basal diet was formulated to meet the nutrient requirements of rabbits [28]. Animal feed was analyzed in triplicate for proximate analyses in accordance with the procedures of AOAC [24]. The chemical analysis of *M. oleifera* and the experimental diets was done at King Saud University, Riyadh, Saudi Arabia. The ingredients and chemical composition of the experimental diets are presented in Table 2.

### 2.3. Growth Performance, Blood Sampling, and Analysis

During the experimental period, all rabbits were weighed at the beginning (0 day) and the end of the trial (42 days). Feed intake of growing rabbits were recorded weekly. Accordingly, daily weight gain, daily feed intake, and feed conversion ratio (FCR) were calculated. At the end of the trial (week 12 of age), blood samples (5 mL/rabbit) were randomly collected into tubes from the ear vein of 14 rabbits (7 males and 7 females as representative sampling) from each group. The blood samples were collected and then serum was prepared by centrifugation for 20 min at 860 g and stored at −20 °C until further analysis. Serum total protein, albumin, glucose, urea, creatinine, uric acid, bilirubin, cholesterol, triglycerides, high-density lipoprotein (HDL), low-density lipoprotein (LDL), alanine aminotransferase (ALT), and aspartate aminotransferase (AST) were measured by ultraviolet spectrophotometer UV4802 (Unico Co., Dayton, OH, USA) using available kits (Biosystem S.A, Costa Brava, 30, Barcelona, Spain) according to the instructions of the manufacturer. The values of serum globulin were determined by subtracting the albumin values from the total protein values. The determination of 2-thiobarbituric acid reactive substances (TBARS) was done using a spectrophotometer, according to the method of Esterbauer and Zollner [29] by detection of malondialdehyde (MDA) in blood using a commercial kit (Sigma-Aldrich St. Louis, MO, USA) following the procedures of the manufacturer. Results were expressed as nmol of MDA/mL. The activity of glutathione peroxidase in blood were also measured using commercial kits (Biodiagnostic, Giza, Egypt) according to the manufacturer instructions and as described by Plaser et al. [30].

### 2.4. Carcass Traits

At the end of the trial (12 weeks of age), 14 rabbits (7 males and 7 females) per group after 12 h of fasting were randomly selected, weighed, and slaughtered into the trial slaughterhouse. The carcasses were chilled and dissected in accordance with the recommendations of Blasco and Ouhayoun [31]. The carcasses of rabbits were weighed, and the slaughter yield was calculated. The weights of the liver, heart, kidney, spleen, and abdominal fat were recorded and stated as % of pre-slaughter weight (index). The length of the cecal appendix and whole intestinal tract (small and large intestine) were also recorded. The cecal pH was determined directly by inserting a pH glass electrode. The longissimus lumborum muscles (LL) were excised from the two sides of the refrigerated carcasses (24 h at 4 °C) and stored at −20 °C until further analyses. 

### 2.5. Physical and Chemical Characteristics of Meat

The pH of LL was recorded in triplicate at 24 h post-mortem with a pH Meter (Beckman model 350, US) as described by Egan et al. [32]. The color parameters of LL were measured in accordance with the Commission International de l’Eclairage [33] using a model CR 410 Chroma meter (Konica Minolta, Tokyo, Japan) and recorded the average of three subsequent readings of lightness (L*), redness (a*) and yellowness (b*) of the LL samples at 24 h post-mortem. For determination of the chemical composition of LL, DM (950.46), CP (981.10), EE (960.39), and ash (920.153) were performed in triplicate in accordance with AOAC [24]. The meat cholesterol contents were measured according to Bohac and Rhee [34] using a spectrophotometer (UV4802, Unico Co., Dayton, OH, USA). The intramuscular lipids were extracted as described by Folch et al. [35]. The determination of the meat FA was performed by the conversion of oil to FA methyl esters followed the procedure described by Yang et al. [36] using a gas chromatograph (Model GC-14A, Shimadzu Corporation, Kyoto, Japan) with a flame ionization detector and a polar capillary column (BPX70, 0.25; SGE Incorporated, USA). The FA methylated esters were recognized by comparing their retention times with those of their standards (Sigma-Aldrich St. Louis MO, US). The FA relative percent was quantified as a percentage of the total analyzed FA in the sample.

### 2.6. Meat Lipid Peroxidation

Meat oxidative stability was determined by evaluating the TBARS content on the meat samples at 3 days after chilling at 4 °C, as described by Ohkawa et al. [37] using commercial kits (Sigma-Aldrich St. Louis, MO, USA), and was expressed as nmol/g of the meat. Meat GPx activity was determined following the method of Paglia and Valentine [38] using commercial kits (Sigma-Aldrich St. Louis, MO, USA), and was expressed as U/g of meat. 

### 2.7. Statistical Analysis

The Kolmogorov Smirnov test was done to determine the normality of the frequency distribution of the data. All variables were subjected to One-way ANOVA using IBM SPSS statistical package (version 22, SPSS Inc., Chicago, IL, USA) to determine the effect of diet, as well as a Tukey’s test (*p* < 0.05). The cage was considered as the experimental unit for the growth performance variables and the rabbit for the carcass traits, blood constituents, and meat quality parameters. Orthogonal polynomials were performed to test linear, quadratic, and cubic effects in response to the incremental dietary levels of MOL in growing rabbits. Significance was declared as *p* < 0.05. All reported values are means ± standard error of the mean (SEM). Spearman rank correlation coefficients were performed to determine the significant correlations between serum cholesterol, MDA, GPx, and their meat concentrations (*p* < 0.05).

## 3. Results

### 3.1. Productive Performance and Carcass Characteristics

Data on the effect of MOL on growth performance and carcass traits are shown in Table 3. There was no statistical difference in the initial live weight among the treatment groups. The final live weight, live weight gain(1533 and 1491.3 g vs. 1301.7 and 1206.3 g, respectively), and daily weight gain (36.5 and 35.51 g/d vs. 30.99 and 28.72 g/d, respectively) of growing rabbits fed diets containing MOL at a level of 10 and 15 g/kg of diet (MOL_1%_ and MOL_1.5%_) were significantly greater (*p* < 0.001) than those fed the CON and MOL_0.5%_ diets; this was supported by a linear response (*p* < 0.001). FCR was improved in the MOL_1%_ and MOL_1.5%_ groups compared to the CON and MOL_0.5%_ groups (2.49 and 2.50 vs. 2.89 and 3.14, respectively; *p* < 0.001, linear, *p* < 0.001). On the other hand, along the trial period, there was no significant effect on the daily feed intakes attributable to the dietary supplementation with MOL. The dressing out percentage, the relative weight of the spleen, and the whole intestinal length were significantly greater (*p* < 0.01) in the MOL_1.5%_ group compared to the other treatment groups; this effect was indicated by a linear response (*p* < 0.01) (Table 3). Dietary MOL supplementation linearly decreased (CON vs. MOL, *p* < 0.001; linear, *p* < 0.001) the abdominal fat content, in particular the MOL_1%_ and MOL_1.5%_ groups (0.90 and 1.03 index, respectively), compared to the CON group (1.22 index) (Table 3). 

### 3.2. Blood Parameters

Table 4 presents the data on several serum biochemical constituents. It shows that the levels of the studied serum metabolites were markedly affected, within a normal range, due to the dietary inclusion of MOL, and particularly, there has been a reduction in the concentrations of ALT, AST, and bilirubin (CON vs. MOL, *p* < 0.01; linear, *p* < 0.01; quadratic, *p* < 0.01), whereas total protein and globulin were increased (CON vs. MOL, *p* < 0.05; linear, *p* < 0.05). On the other hand, the dietary supplementation with MOL did not influence the serum albumin, urea, uric acid, or creatinine concentrations. 

**Figure 1:**
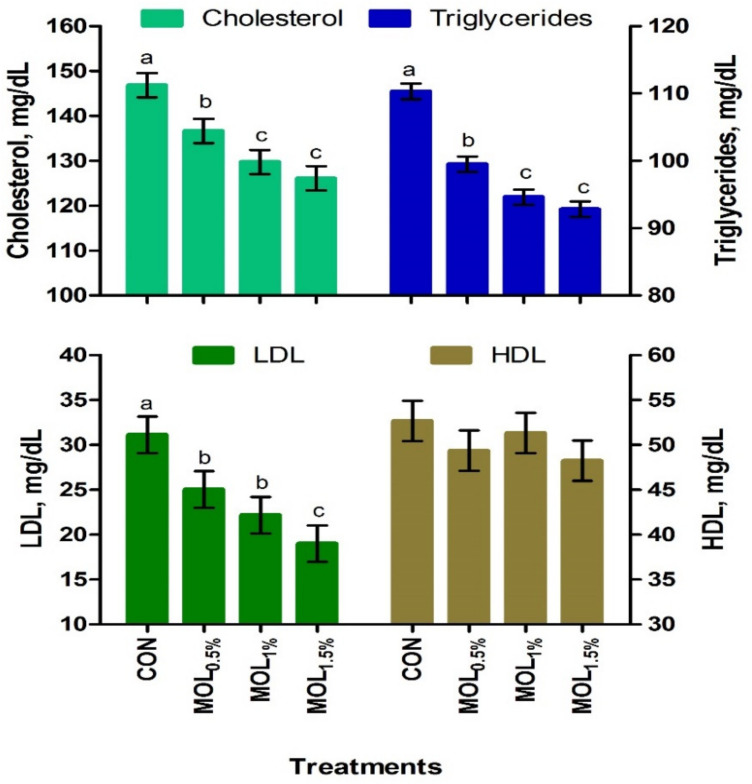
Blood lipid profile of growing rabbits fed the experimental diets. ^a–c^ Means with various letters within each parameter are different at *p* < 0.05. The experimental diets were a basal diet as control (CON) and experimental diets contented 5, 10, or 15 g/kg *Moringa oleifera* leaves (MOL_0.5%,_ MOL_1%,_ and MOL_1.5%_, respectively). Means are presented ± SEM. LDL = low density lipoprotein, HDL = high density lipoprotein.

**Figure 2:**
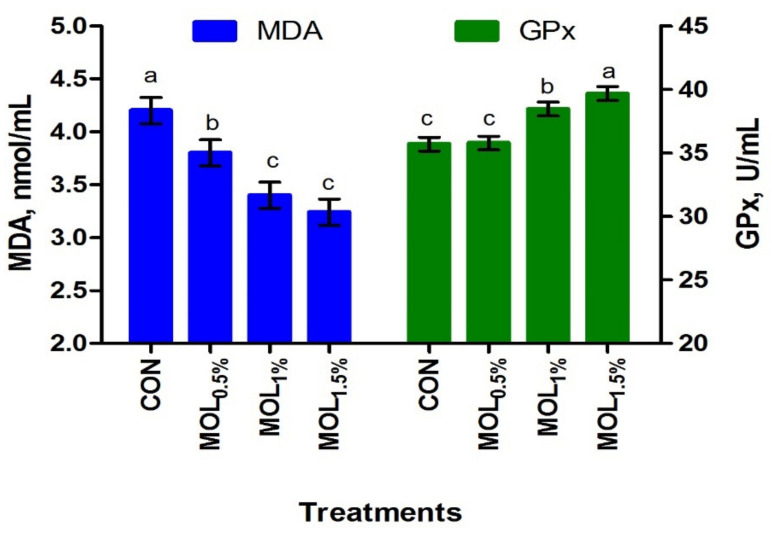
Blood antioxidant capacity of growing rabbits fed the experimental diets. ^a–c^ Means with various letters within each parameter are different at *p* < 0.05. The experimental diets were a basal diet as control (CON) and experimental diets contented 5, 10, or 15 g/kg *Moringa oleifera* leaves (MOL_0.5%,_ MOL_1%,_ and MOL_1.5%_, respectively). Means are presented ± SEM. MDA = malondialdehyde, GPx = glutathione peroxidase.

Figure 1 presents the serum lipid profile of growing rabbits fed the CON and MOL diets. Dietary MOL supplementation for growing rabbits had noticeable linear and quadratic responses regarding the levels of serum total triglycerides (*p* < 0.001 and *p* < 0.05) and cholesterol (*p* < 0.001 and *p* < 0.01), respectively, with lower levels being noticed for growing rabbits consumed the MOL_0.5%,_ MOL_1%_, and MOL_1.5%_ diets compared to those fed the control diet (*p* < 0.001). Furthermore, linear (*p* < 0.001) and quadratic (*p* < 0.01) effects due to the dietary inclusion of MOL were obvious for the serum LDL-cholesterol levels, whereby lower levels of LDL-cholesterol were observed in the MOL-fed rabbits compared with those fed control (*p* < 0.001). Figure 2 shows the levels of MDA and GPx in the serum of the experimental groups. The MDA values were linearly (*p* < 0.001) and quadratically (*p* < 0.01) decreased (*p* < 0.001) in the MOL supplemented groups compared to the CON group, whereas the serum levels of GPx were significantly increased (*p* < 0.001; linear and quadratic, *p* < 0.01) for rabbits fed the MOL diets compared to those fed the CON diet.

### 3.3. Meat Quality Characteristics

Overall, there were no significant changes in pH or L* of LL of rabbits consumed the diets that contained MOL compared with those fed CON (Table 5). The addition of MOL to the diets of growing rabbits linearly increased the a* values (*p* < 0.05) but decreased the b* values (*p* < 0.01) of LL when compared with the CON ones. Dietary MOL supplementation linearly decreased the EE content of LL (*p* < 0.001), while increased the CP content linearly (*p* = 0.001) and quadratically (*p* = 0.01). The concentration of meat cholesterol was significantly lower (*p* < 0.001) for growing rabbits fed the MOL-diets (39.07, 38.63, and 39.90 mg/100g) than the CON ones (43.37 mg/100g) (Table 5); this was indicated by both linear and quadratic responses (*p* < 0.01). Positive correlation (r = 0.739, *p* < 0.01) between serum cholesterol and meat cholesterol content was noticed.

### 3.4. FA Profile and Oxidative Stability of LL

**Figure 3:**
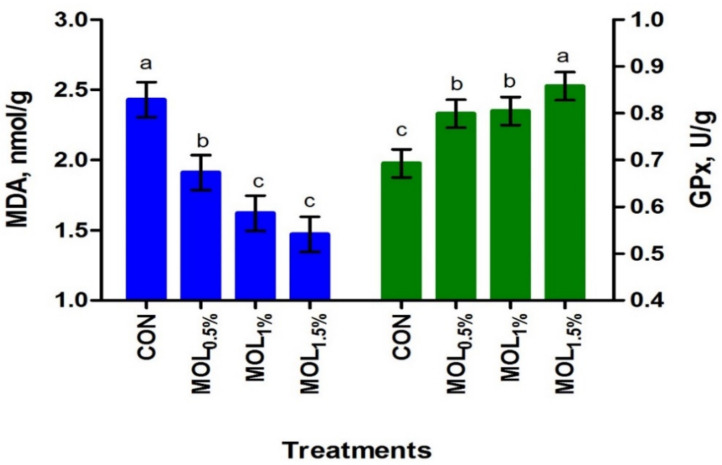
Meat oxidation stability of growing rabbits fed the experimental diets after storage at 4 °C for 72 h. ^a–c^ Means with various letters within each parameter are different at *p* < 0.05. The experimental diets were a basal diet as control (CON) and experimental diets contented 5, 10, or 15 g/kg *Moringa oleifera* leaves (MOL_0.5%,_ MOL_1%,_ and MOL_1.5%_, respectively). Means are presented ± SEM. MDA = malondialdehyde, GPx = glutathione peroxidase.

In LL, the dietary MOL supplementation linearly increased the concentration of PUFA (*p* < 0.001) and linearly and quadratically the n-3 PUFA content (*p* < 0.001), whereas the concentration of SFA decreased (*p* < 0.001; linear, *p* < 0.001; Table 6). The relative content of the meat n-3 PUFA was increased by about 33.71%, 29.46%, and 24.36% for the MOL0.5%, MOL1%, and MOL1.5% groups compared to control. After the storage at 4 ◦C for 72 h, the contents of MDA and GPx in LL were presented in Figure 3. The MDA contents in LL after the storage were linearly and quadratically reduced (*p* < 0.001) due to the dietary MOL supplementation (Figure 3). On the other hand, MOL supplementation in the diets of growing rabbits linearly and quadratically increased (*p* < 0.001) the GPx content in LL when compared to CON. Serum MDA and GPx concentrations showed positive correlations with the LL contents of MDA (r = 0.854, *p* < 0.01) and GPx (r = 0.601, *p* < 0.05), respectively.

## 4. Discussion

Previous studies reported the beneficial impacts of dietary MOL inclusion on the production performance, carcass traits, immunity, and health status of growing rabbits [20,21,22]. However, to the authors’ knowledge, little information is available on the effect of dietary supplementation with MOL on the oxidative stability, physical and chemical characteristics, cholesterol contents, and FA profile of the rabbit’s meat. The nutrients content of MOL used in the current study was in line with previous studies which indicated that MOL is an excellent source of essential nutrients and represents a valuable feed ingredient for growing rabbits [21,22,39].

Our findings showed that the addition of MOL up to 1.5 g/kg in the diets of growing rabbits improved their production performance. These findings are in agreement with earlier studies [20,21,22]. Cui et al. [19] reported an improvement in the growth performance of broiler chickens when MOL was included in their diets at a level of 1.56%. The potential benefits of dietary MOL on the production performance of growing rabbits were observed at a dietary inclusion level of less than 20% of the diet [20,21,22]. The greater live weight gain and better FCR in the present study could be attributed to the bioactive compounds present in MOL [19,21,22], which might have a key role in augmenting the nutrient utilization. It was observed that dietary MOL with lower inclusion levels had a beneficial impact on the rabbit performance, but negative effects were detected by feeding rabbits with higher inclusion levels (30%) of MOL [21,22]. The main cause for lower live weight gain and FCR with high dietary MOL in these previous studies is not obvious and may contribute to an elevated level of several phytochemical components such as phenols, tannins, and phytate [19,21,22].

In the present study, the dietary supplementation with MOL increased the dressing out percentage, which is related to the higher final live weight. Additionally, the increased digestive tract length of rabbits fed MOL in the current study could augment the small intestine absorptive area and nutrient utilization [20,21]. The length and weight of the small intestine were observed to be greater in broiler chickens fed 1.2% MOL [40]. On the contrary, earlier studies recorded that dietary MOL supplementation did not have a noticeable impact on the carcass yield [21,39]. El-Badawi et al. [20] observed that carcass yield was positively increased by a moderate inclusion level (up to 0.45%) of MOL in the diets of growing rabbits. Moreover, the higher dietary inclusion of MOL at a rate of 30% [28] can be efficiently utilized by rabbits without inducing any beneficial impacts on the carcass traits. Greater spleen index (within the normal range) of the rabbits fed the MOL_1.5%_ diet in the current experiment may indicate that MOL could positively impact the immune response of growing rabbits. It has been stated by Sun et al. [21] that MOL addition up to 30% of the rabbit’s diet increased the spleen index and could boost the immune response against pathogens. Isitua and Ibeh [41] observed that rabbits fed MOL showed an increase in CD4 (T-helper) cells and help B-cells and they suggested that MOL could act as a positive immune-regulator. The decreased abdominal fat in rabbits fed MOL in the present study might indicate a difference in lipid metabolism induced by dietary MOL supplementation. It was suggested by Cui et al. [19] that USFA in MOL could augment fatty acid β-oxidation and accordingly reduced abdominal fat deposition. The current findings of spleen and abdominal fat indexes suggested that MOL could beneficially impact the immunity and lipid metabolism of growing rabbits. 

Our data observed that dietary inclusion of MOL positively modifies the meat FA composition, resulting in a reduction of SFA and an increase in PUFA, particularly n-3 FA, with an improvement in the ratio of n-6 to n-3 FA. The higher PUFA content in the meat of rabbits fed the MOL-diets might be owing to the greater concentration of USFA in MOL. Moyo et al. [42] reported that PUFA of MOL represents about 57% of total FA, among which 44.57% of them is α-linolenic acid. Cui et al. [19] found that MOL contained 26.46% α-linolenic acid. Although, type of cultivation, harvest time, method of preparation could influence the FA composition of MOL. Unfortunately, in the current study, we did not analyze the FA composition of MOL to discuss with previous reports. Rabbits can absorb dietary FA and deposit it in the adipose tissue and intramuscular fat, and thus it is likely to modify the meat FA profile through dietary sources of UFA [43]. Unfortunately, there are not any published reports on the effects of feeding MOL on the meat FA profile of growing rabbits to discuss with the findings reported herein. In broiler chickens, Cui et al. [19] observed that dietary inclusion with MOL increased the contents of PUFA, n-3 FA, and n-6 FA in the breast meat. They suggested that a high PUFA content of MOL might be responsible for an elevated PUFA concentration of the breast meat [19]. However, the mechanism of action underlying the modification of meat FA composition in broilers or rabbits fed MOL remains unclear and needs further investigation. In the review by Woods and Fearon [44], they documented that PUFA could deposit straightforward in the meat of non-ruminants through dietary supplementation. The high content of PUFA, in particular n-3 PUFA, and decreased SAF content become a necessary characteristic in the meat products for consumers from the nutritional point of view due to the potential benefits of n-3 PUFA on the immunity and cardiovascular system [45]. The greater concentrations of both PUFA and antioxidants are two significant criteria to consider when a dietary approach is performed.

The meat with a greater PUFA content is always more prone to lipid peroxidation and deterioration, and consequently have a lower shelf-life [46]. Interestingly, in the present trial, dietary MOL addition increased the meat contents of PUFA and GPx but decreased MDA, suggesting that MOL could be a beneficial dietary approach to improve the meat quality of growing rabbits. In addition, there was a positive correlation between the serum and meat MDA contents, as well as, between the serum and meat GPx levels. The enhanced GPx and the diminished MDA levels in both serum and meat supported the idea that MOL could enhance the antioxidant properties and thus resulting in better meat quality [7,19]. Our findings are in line with earlier studies, which reported that dietary inclusion of MOL for broiler chickens [19] and rabbits [22] improved meat oxidative stability during storage. Phenols and potent antioxidants in MOL could be deposited in the meat, exerting a potential diminishing effect on the meat lipid oxidation efficiently by augmenting the antioxidant enzymes and scavenging free radicals [11,12,42]. Little information is available in the literature about the effect of MOL on the meat oxidation stability of growing rabbits. 

In the current study, greater CP and lower EE contents of LL were observed for rabbits fed the MOL diets. These findings suggested that MOL could impact the meat proximate analysis. Sun et al. [21] reported no significant difference in the proximate chemical composition of the rabbit meat fed MOL at a level of 10, 20, and 30% of the diet. There is a paucity of literature on the effects of MOL on the meat nutrient composition of growing rabbits. Meat color is a significant measure of the meat characteristics in meat manufacturing [47]. Dietary inclusion of MOL up to 1.5 g/kg DM improved the meat color after the storage period (72 h), which was supported by an increase in the a ∗ (redness) values and a decrease in the b ∗ (yellowness) values. The augmentation of meat oxidative stability and antioxidant activity due to dietary supplementation with MOL was expected to be responsible for the meat color enhancement. Myoglobin, the sarcoplasmic heme protein, acts as the chief pigment accountable for the red color of the meat, is oxidized during storage, and will cause a change in the meat color [48]. It was reported that a low meat pH decreases the selective absorbance of myoglobin, causing the meat to look less red and more yellow [21,48]. Our results are in agreement with earlier studies in rabbits [21] and broiler chickens [19], which reported that dietary MOL could beneficially impact the redness and yellowness of the meat. 

The reduced cholesterol content in LL of growing rabbits fed the MOL diets might be related to the phenolic compounds present in MOL. These phenolic compounds would lower circulating cholesterol by serving as a natural hypo-cholesterolemic agent and hence a lesser content of meat cholesterol. This was supported by a positive correlation between serum and meat cholesterol concentrations in the current study. Earlier studies suggested that the effect of MOL extract in lowering lipid levels may result from the phenolic compounds, polyphenolic, and flavonoids [49,50]. These bioactive compounds can reduce the uptake of dietary cholesterol from the intestine of rats [49,50]. Polyphenolic compounds were reported to induce a variety of biological properties, involving a reduction of blood lipids due to the greater expression of LDL receptors, diminishing lipid synthesis and lipoprotein secretion in the liver, and augmenting cholesterol removal via bile acids [7,51]. Furthermore, it was assumed that the higher fiber content of MOL may be responsible for lowering blood cholesterol concentration via augmenting lipid metabolism [7,8]. Nevertheless, little information is available about the effect of the dietary inclusion of MOL on the meat cholesterol content in growing rabbits. The latter data can assist to provide additional evidence to the usefulness and safety of MOL as a potential feed ingredient for growing rabbits.

Blood biochemical constituents are considered as an effective method to assess the health status of animals [52]. The absence of a significant effect of MOL on the serum creatinine, urea, bilirubin, or uric acid concentrations indicated that the inclusion levels of MOL up to 15 g/kg of the diet did not induce any adverse effects on the kidney functions of the experimental rabbits. In this regard, *M. oleifera* extract was shown to be effective against gentamicin-induced nephrotoxicity of rabbits due to the detoxification effect of *M. oleifera* extract on the kidney [53]. Greater serum levels of total protein and globulin and lower levels of ALT and AST in the MOL-fed groups in the present study suggested better immunity and liver functions of these rabbits. *M. oleifera* extract was reported to have a hepatoprotective activity due to the presence of bioactive flavonoids such as quercetin [54]. Sun et al. [21] and Salem et al. (22) reported that dietary inclusion of MOL enhanced the blood parameters of growing rabbits. On the contrary, Makanjuola et al. [55] observed that dietary supplementation of MOL up to 0.6% of the diet did not affect the serum total protein, albumin, globulin, and AST concentrations of broiler chickens. Greater serum globulin level of the MOL-fed rabbits supported the immune-stimulant effect of *M. oleifera* since globulins are mainly responsible for enhancing immunity [56]. Our findings are consistent with the results reported by Salem et al. [22].

The addition of MOL in diets of growing rabbits had both linear and quadratic responses regarding the concentrations of serum total triglycerides, LDL, and cholesterol, with lower levels being noticed for rabbits consumed the MOL diets. Consistent with the current findings, Salem et al. [22] observed that the addition of MOL up to 20% in the diets of growing rabbits lowered serum total lipids, cholesterol, triglycerides, and LDL. Jain et al. [49] reported that MOL exerted hypo-lipidemic properties in rats. LDL is a major constituent of the total blood cholesterol and is mainly attributed to the occurrence of cardiovascular diseases and hence, should be considered as a key goal of any lipid-lowering agent, such as MOL, as implied in the present trial. *M. oleifera* is identified as a potent antioxidant feed ingredient due to its antioxidant nutrients, involving vitamins C and E, carotenoids, flavonoids, and selenium, and beneficial phytochemicals [7,8] which might have a crucial role in enhancing the health status of growing rabbits.

## 5. Conclusions

In conclusion, the dietary inclusion of MOL increased final live weight and average daily weight gain, with better FCR without any detrimental effect on animal health status. Dietary MOL supplementation up to 1.5 g/kg DM enhanced the PUFA contents, oxidative stability, CP content, and the meat color, but lowered the meat EE content and abdominal fat index; this was supported by linear and quadratic responses. The inclusion of MOL in the diet of growing rabbits decreased blood lipids, including TAG, cholesterol, and LDL, whereas improved blood antioxidant capacity (low MDA and greater GPx). These results suggested that MOL could be used as a feed supplement at a level of 1.5 g/kg of the diet for growing rabbits with beneficial impacts on the rabbit health, growth performance, and the nutritional value of the meat.

## Figures and Tables

**Table 1:** Nutrients and phenolic acids content of *M. oleifera* leaves (MOL) as DM basis.

Item ^a^	MOL
Proximate analysis, %	
DM	91.0
CP	27.3
EE	6.96
CF	11.02
NDF	16.80
ADF	12.79
Ash	14.2
Ca	2.08
P	0.35
Lysine	1.70
Methionine	0.42
Vit. A, mg/kg	4.50
Vit. C, mg/kg	7.30
Vi. E, mg/kg	27.0
Phenolic acids, mg/100 g	
Chlorogenic acid	5.39
Ferulic acid	32.90
Caffeic acid	53.32
Rutin	75.02
Quercetin	3.74
Gallic acid	12.30
Total phenolics	2089 mg gallic acid equivalent/100 g
Total flavonoids	46.53 mg quercetin equivalent/g

^a^ DM = dry matter, CP = crude protein, EE = ether extract, CF = crude fiber, NDF = neutral detergent fiber, ADF = acid detergent fiber, Ca = calcium, P = phosphorus.

**Table 2:** Ingredients and chemical composition of the experimental diets (g/kg) as DM basis.

Items	Experimental Diets ^a^			
	CON	MOL_0.5%_	MOL_1%_	MOL_1.5%_
Ingredients				
Berseem hay	370	365	360	355
Yellow corn	175	175	175	175
Soybean meal, 44%	170	170	170	170
Barley	100	100	100	100
Wheat bran	140	140	140	140
MOL	0	5	10	15
Molasses	20	20	20	20
Limestone	10	10	10	10
Dicalcium Phosphate	7	7	7	7
Common salt	5	5	5	5
Premix ^b^	3	3	3	3
Total	1000	1000	1000	1000
Chemical composition, analyzed values				
DM	899	907	897	910
CP	181.6	182.8	183.2	183.9
EE	25.2	25.4	25.4	25.4
CF	135.0	134.0	133.2	133.3
NDF	317.7	315.4	314.4	313.0
ADF	197.0	195.5	194.2	193.0
Ash	61.7	62.0	62.2	62.6
DE, MJ/kg	11.22	11.24	11.25	11.26

^a^ The experimental diets were a basal diet as control (CON) and experimental diets contented 5, 10, or 15 g/kg *Moringa oleifera* leaves (MOL_0.5%,_ MOL_1%,_ and MOL_1.5%_, respectively). ^b^ Supplied per kg of ration: vit. A 12,000 IU; vit. D_3_ 2000 IU; vit. E 50 mg; vit. B_1_ 2.5 mg; vit. B_2_ 4 mg; vit. B_6_ 1.25 mg; vit. B_12_ 0.01 mg; vit. K 2.5 mg; niacin 30 mg; folic acid 5 mg; pantothenic acid 15 mg; choline 1200 mg; Mn 60 mg; Cu 3 mg; Fe 50 mg; Zn 60 mg.

**Table 3:** Growth performance and carcass traits of growing rabbits fed the control and treatment diets.

Items	Treatments ^a^				SEM	*p*-Value			
	CON	MOL_0.5%_	MOL_1%_	MOL_1.5%_		Treatment Effect	Linear	Quadratic	Cubic
Growth performance ^b^									
Initial LW, g	960.4	956.4	911.9	965.2	35.3	0.42	--	--	--
Final LW, g	2166.6 ^b^	2258.1 ^b^	2403.3 ^a^	2498.2 ^a^	34.9	<0.001	<0.001	0.94	0.36
LWG, g	1206.3 ^b^	1301.7 ^b^	1491.3 ^a^	1533.0 ^a^	49.6	<0.001	<0.001	0.43	0.15
Daily LWG, g/d	28.72 ^b^	30.99 ^b^	35.51 ^a^	36.50 ^a^	1.42	<0.001	<0.001	0.43	0.15
Daily FI, g/d	90.17	89.14	88.77	90.88	1.33	0.22	0.49	0.62	0.61
FCR	3.14 ^a^	2.89 ^a^	2.50 ^b^	2.49 ^b^	0.09	<0.001	<0.001	0.09	0.11
Carcass characteristics									
Dressing, %	58.23 ^b^	58.61 ^b^	59.15 ^b^	61.74 ^a^	0.56	0.001	<0.001	0.04	0.30
Abdominal fat, %	1.22 ^a^	1.03 ^b^	1.03 ^b^	0.90 ^c^	0.04	<0.001	<0.001	0.43	0.84
Liver, %	3.82	3.84	3.80	3.86	0.56	0.58	0.57	0.46	0.29
Spleen, %	0.08 ^b^	0.09 ^ab^	0.12 ^ab^	0.14 ^a^	0.02	0.02	0.004	0.67	0.58
Heart, %	0.30	0.28	0.31	0.30	0.01	0.49	0.75	0.56	0.74
Kidney, %	0.41	0.39	0.42	0.41	0.12	0.18	0.46	0.58	0.50
Cecal appendix, cm	10.48	10.15	10.64	10.62	0.35	0.51	0.44	0.55	0.26
Intestinal length, cm	355 ^b^	356 ^b^	366 ^ab^	375 ^a^	10.2	0.03	0.008	0.84	0.43
Duodenal pH	6.80	6.80	6.88	6.73	0.13	0.87	0.80	0.47	0.52
Cecal pH	7.17	7.16	7.18	7.11	0.09	0.84	0.54	0.56	0.77

^a–c^ Means with various superscripts within each parameter are different at *p* < 0.05; Tukey’s test was performed to compare means; SEM = Standard error of the mean. ^a^ The experimental diets were a basal diet as control (CON) and experimental diets contented 5, 10, or 15 g/kg *Moringa oleifera* leaves (MOL_0.5%_, MOL_1%_, and MOL_1.5%_, respectively). ^b^ LW = live weight, LWG = live weight gain, FI = feed intake, FCR = feed conversion ratio.

**Table 4:** Blood biochemical constituents of growing rabbits fed the experimental diets.

Items ^b^	Treatments ^a^				SEM	*p*-Value			
	CON	MOL_0.5%_	MOL_1%_	MOL_1.5%_		Treatment Effect	Linear	Quadratic	Cubic
TP, g/dL	6.10 ^b^	6.24 ^b^	6.47 ^ab^	6.70 ^a^	0.13	0.04	0.03	0.51	0.11
Alb., g/dL	3.50	3.33	3.38	3.30	0.42	0.66	0.51	0.43	0.79
Globulin, g/dL	2.50 ^c^	2.91 ^b^	3.09 ^b^	3.40 ^a^	0.12	0.02	0.04	0.70	0.13
ALT, U/L	64.73 ^a^	62.33 ^b^	62.43 ^b^	60.63 ^c^	0.12	˂0.001	˂0.001	0.001	0.15
AST, U/L	19.63 ^a^	16.53 ^b^	16.73 ^b^	16.60 ^b^	0.21	˂0.001	˂0.001	˂0.001	0.12
Urea, mg/dL	42.01	41.02	41.50	40.30	0.54	0.06	0.03	0.80	0.09
Uric acid, mg/dL	0.168	0.163	0.160	0.161	0.005	0.19	0.06	0.26	0.82
Creatinine, mg/dL	1.10	1.04	1.06	1.09	0.04	0.40	0.93	0.12	0.56
Bilirubin, mg/dL	1.04 ^a^	0.93 ^b^	0.85 ^b^	0.82 ^b^	0.05	0.008	0.001	0.32	0.90

^a–c^ Means with various superscripts within each parameter are different at *p* < 0.05; Tukey’s test was performed to compare means; SEM = Standard error of the mean. ^a^ The experimental diets were a basal diet as control (CON) and experimental diets contented 5, 10, or 15 g/kg *Moringa oleifera* leaves (MOL_0.5%,_ MOL_1%,_ and MOL_1.5%_, respectively). ^b^ TP = total protein, Alb = albumen, ALT = alanine aminotransferase, AST = aspartate aminotransferase.

**Table 5:** Meat physical and chemical characteristics of growing rabbits fed the experimental diets.

Items	Treatments ^a^				SEM	*p*-Value			
	CON	MOL_0.5%_	MOL_1%_	MOL_1.5%_		Treatment Effect	Linear	Quadratic	Cubic
Physical characteristics									
pH	5.82	5.79	5.72	5.76	0.12	0.51	0.65	0.56	0.84
Color ^b^									
L *	56.40	56.71	55.93	56.50	0.57	0.77	0.95	0.86	0.32
a *	10.61 ^b^	11.65 ^a^	11.83 ^a^	11.75 ^a^	0.34	0.03	0.02	0.13	0.35
b *	4.30 ^a^	3.90 ^ab^	3.50 ^b^	3.03 ^b^	0.17	0.006	0.01	0.45	0.35
Chemical composition									
moisture, %	74.76	74.34	74.57	74.42	0.67	0.48	0.63	0.80	0.38
CP, %	20.46 ^c^	21.27 ^b^	21.62 ^a^	21.74 ^a^	0.13	˂0.001	0.001	0.01	0.50
EE, %	3.41 ^a^	2.84 ^b^	2.48 ^c^	2.64 ^c^	0.12	˂0.001	0.001	0.23	0.11
Ash, %	1.37	1.49	1.33	1.20	0.16	0.66	0.67	0.83	0.27
Cholesterol, mg/100g	43.37 ^a^	39.90 ^b^	38.63 ^b^	39.07 ^b^	0.67	˂0.001	˂0.001	0.003	0.82

^a–c^ Means with various superscripts within each parameter are different at *p* < 0.05; Tukey’s test was performed to compare means; SEM = Standard error of the mean. ^a^ The experimental diets were a basal diet as control (CON) and experimental diets contented 5, 10, or 15 g/kg *Moringa oleifera* leaves (MOL_0.5%,_ MOL_1%,_ and MOL_1.5%_, respectively). ^b^ L * = lightness, a * = redness, b * = yellowness.

**Table 6:** Meat FA profile (% of total FA) of growing rabbits fed the experimental diets.

Items ^b^	Treatments ^a^				SEM	*p*-Value			
	CON	MOL_0.5%_	MOL_1%_	MOL_1.5%_		Treatment Effect	Linear	Quadratic	Cubic
∑SFA	41.52 ^a^	40.33 ^b^	39.80 ^b^	37.95 ^c^	0.23	˂0.001	˂0.001	0.07	0.02
∑MUFA	28.54	28.43	28.37	29.60	0. 96	0.21	0.26	0.24	0.69
∑PUFA	29.94 ^b^	31.23 ^b^	31.83 ^b^	32.45 ^a^	0.30	˂0.001	˂0.001	0.46	0.10
∑n-3 FA	3.53 ^c^	4.39 ^b^	4.57 ^ab^	4.72 ^a^	0.07	˂0.001	˂0.001	0.001	0.01
∑n-6 FA	26.41	26.84	27.26	27.73	0.50	0.20	0.31	0.42	0.30
n-6:n-3	7.48 ^a^	6.11 ^b^	5.96 ^b^	5.88 ^b^	0.15	˂0.001	˂0.001	0.001	0.06
PUFA:SFA	0.75 ^b^	0.77 ^b^	0.78 ^b^	0.83 ^a^	0.01	˂0.001	˂0.001	0.14	0.04
UFA	58.48 ^c^	59.67 ^b^	60.20 ^b^	62.05 ^a^	0.23	˂0.001	˂0.001	0.07	0.02

^a–c^ Means with various superscripts within each parameter are different at *p* < 0.05; Tukey’s test was performed to compare means; SEM = Standard error of the mean. ^a^ The experimental diets were a basal diet as control (CON) and experimental diets contented 5, 10, or 15 g/kg *Moringa oleifera* leaves (MOL_0.5%,_ MOL_1%,_ and MOL_1.5%_, respectively). ^b^ SFA = saturated fatty acids, MUFA = monounsaturated fatty acids, FA = fatty acids, PUFA = polyunsaturated fatty acids, UFA = unsaturated fatty acids.

## Data Availability

The data presented in this study are available on request from the corresponding author.
